# Quantitative Estimation of Auxin, Siderophore, and Hydrogen Cyanide Production in Halo and Drought-Tolerant Bacterial Isolates for Cucumber Growth

**DOI:** 10.21769/BioProtoc.5471

**Published:** 2025-10-05

**Authors:** Zeinab Fotoohiyan, Ali Salehi Sardoei

**Affiliations:** 1 Department of Plant Pathology, Ji.c., Islamic Azad University, Jiroft, Iran; 2 Department of Horticultural and Crops Research, Southern Kerman Agricultural and Natural Resources Research and Education Center, AREEO, Jiroft, Iran

**Keywords:** Auxin, Growth stimulating factor, Hydrogen cyanide, Siderophore, Phosphate solubilization, Cucumber seedling, Resistant bacteria

## Abstract

Salt-tolerant bacteria can enhance plant growth through various mechanisms, including the production of auxin, siderophores, hydrogen cyanide, and the solubilization of insoluble phosphate. This study investigated the production of these growth-stimulating factors by salt- and drought-tolerant bacteria isolated from the arid and saline farmlands of Jiroft. Initially, we screened for bacterial strains that exhibited the highest levels of these factors. We then evaluated their effects on improving the growth indices of cucumber seedlings. Additionally, we optimized the protocols for isolating auxin, siderophores, hydrogen cyanide, and phosphate solubilization, which can also be applied to other host rhizobacteria to assess their growth-promoting compounds.

Key features

• The most resistant bacterial isolates to salinity and drought are identified by adding salt and polyethylene glycol to the culture medium in laboratory conditions.

• This protocol can be used to evaluate the production levels of IAA, siderophore, hydrogen cyanide, and phosphate solubilization by salt- and drought-tolerant bacteria.

• This protocol can also be used to evaluating the plant growth–promoting ability of salt- and drought-tolerant bacteria under greenhouse conditions.

• This protocol can be applied to other host rhizobacteria to assess their growth-promoting compounds.

## Graphical overview


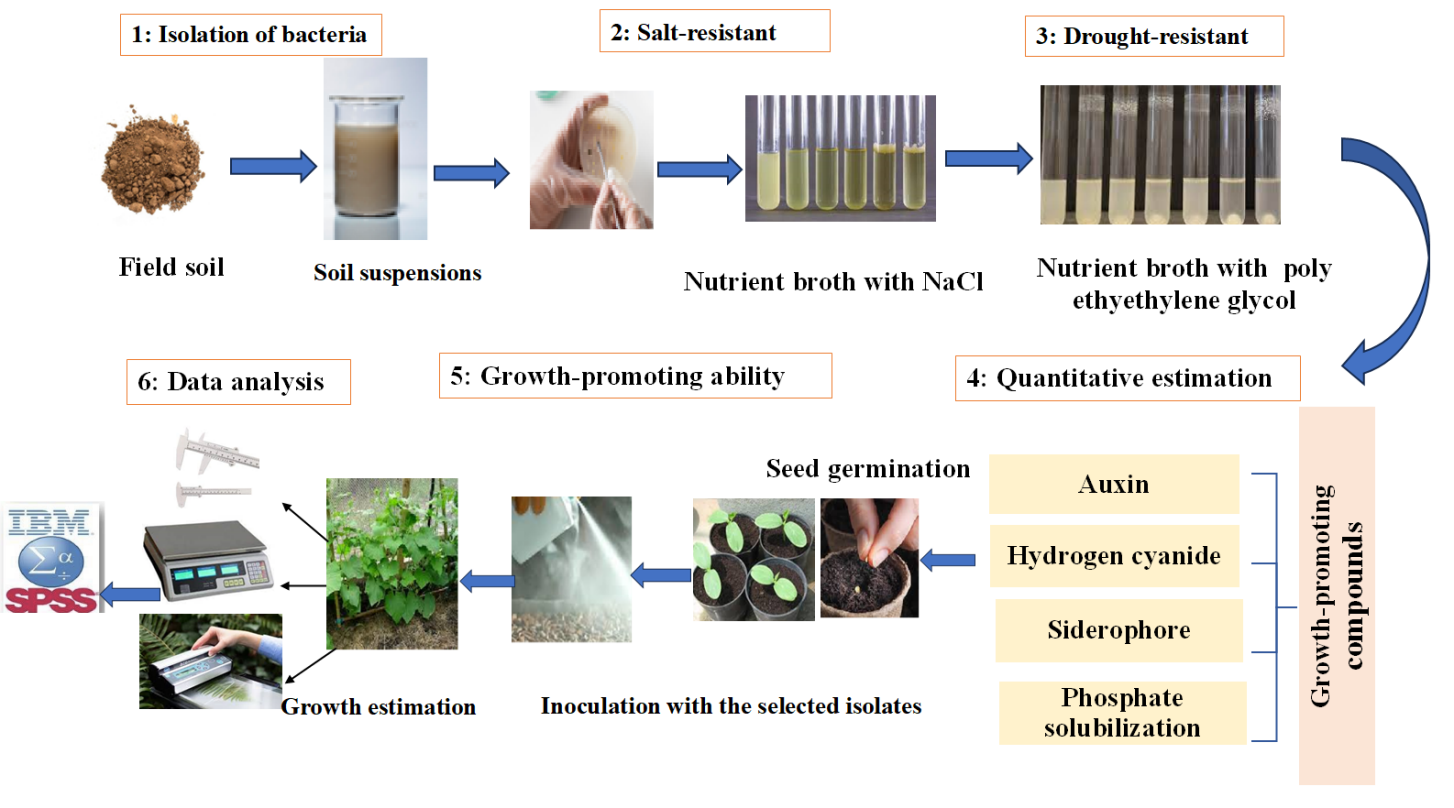


**Graphical representation of the protocol for quantitative estimation of growth-promoting compounds in halotolerant bacterial isolates for cucumber growth.** 1) Isolation of bacteria. 2) Screening for salt-resistant bacteria. 3) Screening for drought-resistant bacteria. 4) Quantitative estimation of auxin, siderophore, and hydrogen cyanide production, as well as phosphate solubilization activity. 5) Assessing the effect of superior isolates on cucumber seedling growth. 6) Data analysis. Arrows indicate the sequence of the experimental procedure.

## Background

In recent years, the use of plant growth–promoting rhizobacteria (PGPR) has emerged as a form of biofertilizer [1]. These bacteria enhance plant resistance to environmental stresses and improve growth and yield through various mechanisms, including nitrogen fixation, solubilization of insoluble phosphate, production of siderophores, synthesis of plant hormones like auxin, and reduction of ethylene concentration [2]. Indole-3-acetic acid (IAA) is one of the most active plant hormones. It plays a crucial role in cell division and elongation, enhances the surface area and weight of root hairs, and promotes the development of lateral roots [3]. In young stems, IAA helps in the apical dominance and stimulates growth [4]. Moreover, IAA-producing PGPRs are reported to boost nutrient uptake and chlorophyll content [5]. Siderophores produced by plant growth–promoting rhizobacteria (PGPR) in response to iron deficiency enhance iron uptake by solubilizing and chelating iron. They form ferric–siderophore complexes that increase iron availability, even at very low concentrations, thereby supporting iron supply and promoting plant growth [6]. According to certain study findings, different bacteria, like *Pseudomonas* and *Bacillus*, can produce siderophores [7-10]. Using the CAS (Chrome-Azurol S) method for initial selection of potential iron-solubilizing isolates is an effective screening method [11]. Moreover, hydrogen cyanide (HCN), produced by many rhizobacteria [12], may affect plant establishment or inhibit the development of plant disease [13]. Some studies suggest that HCN participates in geochemical processes within the substrate, such as metal chelation, which indirectly increases nutrient availability for both rhizobacteria and their plant hosts [14]. Phosphate-solubilizing activity has been reported in many PGPR genera [15]. It is estimated that about 95%–99% of phosphorus in soil is unavailable to plants [16], so the use of such bacteria can replace phosphate fertilizers. Reports indicate that *Bacillus* species enhance plant growth significantly by producing phytohormones and siderophores, as well as by solubilizing phosphate. This results in increased stem length and total dry weight [17]. Bacteria isolated from extremely saline and hot soils by Gil et al. [18] have been shown to enhance root size under osmotic stress in *Medicago* sp. Given the widespread presence of saline soils in Iran and the significant agricultural importance of Jiroft County, this study aims to evaluate the production levels of IAA, siderophore, hydrogen cyanide, and phosphate solubilization by salt- and drought-tolerant bacteria. Additionally, it will assess the impact of these bacteria on the growth of cucumber seedlings. This research marks the first investigation in Jiroft County—the agricultural hub of Iran—focused on promoting the growth of the region’s key agricultural product. If the results are positive, these findings could potentially be applied as a biological fertilizer at the farm level.

## Materials and reagents

**Biological materials**

1. Halotolerant PGPR bacterial strains

*Note: Our strains were isolated from field soil samples at a depth of 30 cm in the arid and saline regions of Jiroft (Kanarsandal, Jazsaleh, Anbarabad, and Karimabad areas) in Kerman province, Iran. All isolates were found to belong to the* Bacillus *genus, with isolate C8 identified as the most effective. Following PCR amplification of the 16S rDNA, an approximate 1,500 bp band was observed on an agarose gel. Amplicon sequencing was conducted by Bioneer Corporation in South Korea. After sequencing the amplified products, BLAST alignment was performed on NCBI to find a strain with a high degree of homology. The molecular weight of these sequence fragments matched that of the bacterial 16S rDNA fragment. Based on the BLAST results and the phylogenetic tree analysis, the C8 isolate was identified as* Bacillus subtilis, *as its amplicon sequence shared 99%–100% similarity with* Bacillus subtilis.

Bacterial codes: K: Kanarsandal; C: Karimabad; J: Jazsaleh; and A: Anbarabad.

2. Cucumber seeds (Royal cultivar) were obtained from the Agricultural and Natural Resources Research Center of Southern Kerman (Iran)

**Reagents**

1. Glycine (Sigma-Aldrich, catalog number: 56-40-6)

2. L-tryptophan (Sigma, catalog number: 65072-00-6)

3. Casamino acids (Sigma, catalog number: 65072-00-6)

4. K_2_HPO_4_ (Sigma, catalog number: 7758-11-4)

5. MgSO_4_·7H_2_O (Sigma, catalog number: 10034-99-8)

6. Glycerin (Sigma, catalog number: 0770525)

8. Tryptone (OXOID, catalog number: LP0042)

9. Agar (Sigma-Aldrich, catalog number: 9002-18-0)

10. Potassium chloride (KCl) (Merck, catalog number: 1.93238.0521)

11. Sodium chloride (NaCl) (Merck, catalog number: 1.93606.0521)

12. Ammonium sulfate [(NH_4_)_2_SO_4_] (Sigma, catalog number: 7783-20-2)

13. Yeast extract (OXOID, catalog number: LP0021)

14. Tricalcium phosphate [Ca_3_(PO_4_)_2_] (Sigma, catalog number: 7758-87-4)

15. Glucose (Sigma, catalog number: 492-62-6)

16. Iron sulfate (FeSO_4_·7H_2_O) (Sigma-Aldrich, catalog number: 7782-63-0)

17. Manganese sulfate (MnSO_4_·H_2_O) (Sigma-Aldrich, catalog number: 10034-96-5)

18. FeCl3 (Sigma, catalog number: 7705-08-0)

19. Chrome azurol S (CAS) (Sigma, catalog number: 1667-99-8)

20. HTDMA (Sigma, catalog number: 10424-65-4)

21. Piperazine solution (Merck, catalog number: 807325)

22. Perchloric acid (HClO_4_) (Sigma, catalog number: 1005190510)

23. Soytone (Sigma, catalog number: 91079-46-8)

24. 2,2′ dipyridyl (DIP) (Sigma, catalog number: 366-18-7)

25. Beef extract (Sigma, catalog number: 68990-90-0)

26. Peptone (Sigma, catalog number: 100209-45-8)

27. Sodium hypochlorite (Nice, catalog number: S2195)

28. Ethanol (Biopack, catalog number: 2000165400)

29. KOH (Sigma, catalog number: 497-19-8)

30. Sterile distilled water

31: Sodium carbonate (Sigma, catalog number: 65072-00-6)

32. Picric acid (Sigma, catalog number: 501840460)

33. Polyethylene glycol (Sigma, catalog number: 25322-68-3)

34. KH_2_PO_4 _(Merck, catalog number: 1.93205.0521)

35. Minimal agar medium (Sigma, catalog number: 45-79332-500G-F)

**Solutions**

1. Nutrient broth medium (see Recipes)

2. Nutrient agar (NA) (see Recipes)

3. Iron (III) solution (see Recipes)

4. MKB medium (see Recipes)

5. Minimal agar medium (see Recipes)

6. Minimum iron medium (see Recipes)

7. Tryptic soy agar (TSA) medium (see Recipes)

8. Picosky solid culture medium (see Recipes)

9. CAS solution (see Recipes)

10. Salkowski reagent (see Recipes)

11. Ethanol 70% (see Recipes)

**Recipes**

**1. Nutrient broth (NB) (1 L)**

**Table:** 

Reagents	Final concentration	Quantity or volume
Beef extract	1.0% (w/v)	1.0 g
Peptone	1% (w/v)	1 g
Yeast extract	2.0% (w/v)	2.0 g
NaCl	0.5% (w/v)	0.5 g
Sterile distilled water	n/a	1 L

Final pH 7.

**2. Nutrient agar (NA) (1 L)**

**Table:** 

Reagents	Final concentration	Quantity or volume
Nutrient broth	8% (w/v)	8 g
Agar	15% (w/v)	15 g
Sterile distilled water	n/a	1 L

**3. Iron (III) solution**

**Table:** 

Reagents	Final concentration	Quantity or volume
FeCl_3_·6H_2_O	1 mmol/L	1 mmol
HCl	10 mmol/L	10 mmol
Sterile distilled water	-	1 L

**4. MKB medium (1 L)**

**Table:** 

Reagents	Final concentration	Quantity or volume
K2HPO4	2.5 g/L	2.5 g
MgSO_4_·7H_2_O	2.5 g/L	2.5 g
Glycerin	15 mL/L	15 mL/L
Casamino acids	5.0 g/L	5.0 g
Sterile distilled water	-	1 L

Final pH 7.2.

**5. Minimal agar medium (1 L)**

**Table:** 

Reagents	Final concentration	Quantity or volume
Dextrose	1% (w/v)	1 g
Dipotassium phosphate	7% (w/v)	7 g
Monopotassium phosphate	2% (w/v)	2 g
Sodium citrate	0.5% (w/v)	0.5 g
Magnesium sulphate	0.1% (w/v)	0.1 g
Ammonium sulphate	1% (w/v)	1 g
Agar	15% (w/v)	15 g
Sterile distilled water	-	1 L

Final pH 7.

**6. Minimum iron medium (1 L)**

**Table:** 

Reagents	Final concentration	Quantity or volume
Tryptone	0.1%	0.1 mL
Casamino acids	0.1%	0.1 mL
Tryptophan (CAS+Trp(	0.1 mg/mL	0.1 mg
Yeast extract	0.1%	0.1 mL
DIP	0.1%	0.1 mL
Sterile distilled water	-	1 L

Final pH 6.85.

**7. Tryptic soy agar (TSA) medium (1 L)**

**Table:** 

Reagents	Final concentration	Quantity or volume
Tryptone	15% (w/v)	15 g
Soytone	5% (w/v)	5 g
NaCl	5% (w/v)	5 g
Agar	5% (w/v)	5 g
Sterile distilled water	n/a	1 L

**8. Picosky solid culture medium (1 L)**

**Table:** 

Reagents	Final concentration	Quantity or Volume
Glucose	10% (w/v)	10 g
Tricalcium phosphate	5% (w/v)	5 g
Yeast extract	0.5% (w/v)	0.5 g
Ammonium sulfate	0.5% (w/v)	0.5 g
Potassium chloride	0.2% (w/v)	0.2 g
Sodium chloride	0.2% (w/v)	0.2 g
Magnesium sulfate	0.1% (w/v)	0.1 g
Iron sulfate	0.0003% (w/v)	0.0003 g
Manganese sulfate	0.0003% (w/v)	0.0003 g
Agar	10% (w/v)	10 g
Sterile distilled water	n/a	1 L

Final pH 7.

**9. CAS solution**

**Table:** 

Reagents	Final concentration	Quantity or volume
1 mM FeCl_3_	1.5%	1.5 mL
1 mM CAS	7.5%	7.5 mL
4 mmol/L HTDMA	50%	50 mL
1 M piperazine solution	30%	30 mL
Sterile distilled water	n/a	100 mL

**10. Salkowski reagent**

**Table:** 

Reagents	Final concentration	Quantity or volume
35% perchloric acid (HClO_4_)	49%	49 mL
0.5 M FeCl_3_	1%	1 mL

**11. Ethanol 70%**

**Table:** 

Reagents	Final concentration	Quantity or volume
Ethanol 96%	73%	73 mL
Sterile distilled water	27%	27 mL

**Laboratory supplies**

1. Sterile glass rod

2. Petri dishes, 90 mm (Abdos, catalog number: 3165077)

3. Microtips 200–1,000 μL (Tarson, catalog number: 521020)

4. Microtips 2–200 μL (Tarson, catalog number: 521010)

5. Microtips 0.2–10 μL (Tarson, catalog number: 521000)

6. Culture bottles (Tarsons, catalog number: 20080)

7. Fungicide captain (Wp50%)

8. Clean pots (21 × 16 × 16 cm)

9. Micropipettes (Eppendorf Research Plus, catalog number: 35016146026)

10. 2 mL microcentrifuge tubes (Eppendorf, catalog number: P10203)

11. 50 mL centrifuge tubes (Falcon, catalog number: 546024)

12. Filter papers (Whatman No. 1) (Sigma, catalog number: FP/310/AC)

13. Cotton (HiMedia, catalog number: LA1018)

14. Parafilm (Amcor, catalog number: PM-996)

15. Perlite (Keltech Energies Limited, catalog number: 27030090)

16. Household aluminum foil for multiple uses

17. 5 mL microcentrifuge tubes (Eppendorf, catalog number: 4092.4N)

## Equipment

1. Spectrophotometer (Jenway, model: 6505)

2. Rotary shaker incubator (Biological Oxygen Demand, BOD)

3. Digital camera (Canon, model: DS126431)

4. mm-precision graduated ruler

5. Digital scale with accuracy of 0.001 g

6. Oven (DIAL 2.5 AMPS 240 V 600 W, catalog number: BARN3606-1CE)

7. Leaf scanner (Hewlett-Packard Scan Jet 7400C scanner)

8. Vortexer (VWR^®^ Fixed Speed Vortex Mixer, catalog number: 10153-834)

9. Centrifuge (Eppendorf 5424R Microcentrifuge, catalog number: 05-401-205)

## Software and datasets

1. ImageJ software (NIH, https://imagej.net/)

2. Excel (Microsoft Office 2016)

3. Photoshop graphical software (2025) (v. 26.10.0.7)

4. SPSS Statistics Software Version 26

## Procedure

**A. Bacterial isolation**

1. Collect soil samples from a depth of 30 cm.

Note: We collected soil from the dry and saline areas of Jiroft County farms (Kanarsandal, JozeSaleh, Anbarabad, and Karimabad) in Kerman Province, Iran, during the 2020 agricultural year.

2. Transfer samples to the laboratory in sterile plastic bags.

3. Add 10 g of each soil sample to vials containing 90 mL of sterile distilled water.

4. Shake the mixture at 100 rpm and 30 °C for 1 h.

5. Prepare serial dilutions of the soil suspensions up to 10^-5^.

6. Prepare minimal agar medium.

7. Prepare TSA medium in a slant form in screw-cap test tubes.

8. Perform spread plating using a glass rod, adding 60 μL of each dilution to the minimal agar medium.

9. Incubate the samples at 30 °C in the dark for 48 h. Randomly select five isolates per rhizosphere soil sample and re-streak them on TSA plates for colony purification.

10. Culture all purified isolates on TSA in test tubes to prepare stock cultures.

11. Incubate for 24 h at 30 °C.

12. Store the isolates in a refrigerator at 4 °C.

**B. Screening of salt-tolerant bacteria**

1. Prepare nutrient broth liquid medium.

2. Add sodium chloride in varying concentrations of 0, 5, 10, 15, 20, 25, 35, and 40 g to create salinity levels of 5%, 10%, 15%, 20%, 25%, 35%, and 40%, respectively. Adjust the total volume in each flask to 100 mL with nutrient broth.

3. Incubate the samples for 24 h at 30 °C.

4. Measure the growth of the isolates using a spectrophotometer, with optical density readings taken at 660 nm.

5. Select 11 salt-tolerant isolates capable of growing in a medium containing 40% sodium chloride.

**C. Screening of drought-tolerant bacteria**

1. Create water potentials of 0.50, -0.15, -0.30, -0.49, and -0.73 MPa by adding calculated amounts of polyethylene glycol 6000 to each liter of nutrient broth medium, following the formula below [19]:

Water potential (wp) = –(1018e-2)c – (1.18e-4)c + (2.67e-4)ct + (8.39e-7)cT

Where:

T = temperature in Kelvin

c = polyethylene glycol concentration

t = ambient temperature

e = constant coefficient

wp = water potential

2. Add a 0.1 mL sample of liquid culture containing salt-tolerant bacteria to each of the different water potential treatments, with three replicates for each treatment.

3. Incubate the vials at 30 ± 2 °C on a shaker set to 120 rpm for 24 h.

4. Assess bacterial growth by measuring optical density at 660 nm using a spectrophotometer.

5. Prepare control samples without the addition of polyethylene glycol.

**D. Quantitative estimation of auxin**

1. Estimate auxin levels using the Brick method [20].

2. Prepare a nutrient broth medium containing 0.5% L-tryptophan (500 μg/mL) and 2% sodium chloride

3. Culture the isolates in this medium (Figure 1.1).

4. Incubate the cultures in a shaking incubator at 27 ± 2 °C for 48 h (Figure 1.2).

5. After incubation, centrifuge the samples for 10 min at 11,200rpm (Figure 1.3).

6. Prepare Salkowski reagent (see Recipe 9).

7. Mixed 1 mL of the supernatant with 1 mL of Salkowski reagent in a test tube (Figure 1.4).

8. Incubate the reaction mixture at 27 ± 2 °C for 20 min in the dark.

9. The presence of IAA is indicated by a pinkish-red color in the test tube (Figure 1.5–6).

10. Measure the optical absorption at 530 nm (Figure 1.7).

11. Estimate the amount of IAA using a standard IAA curve (Hi-media) and expressing in μg/mL [21].

**Figure 1.**
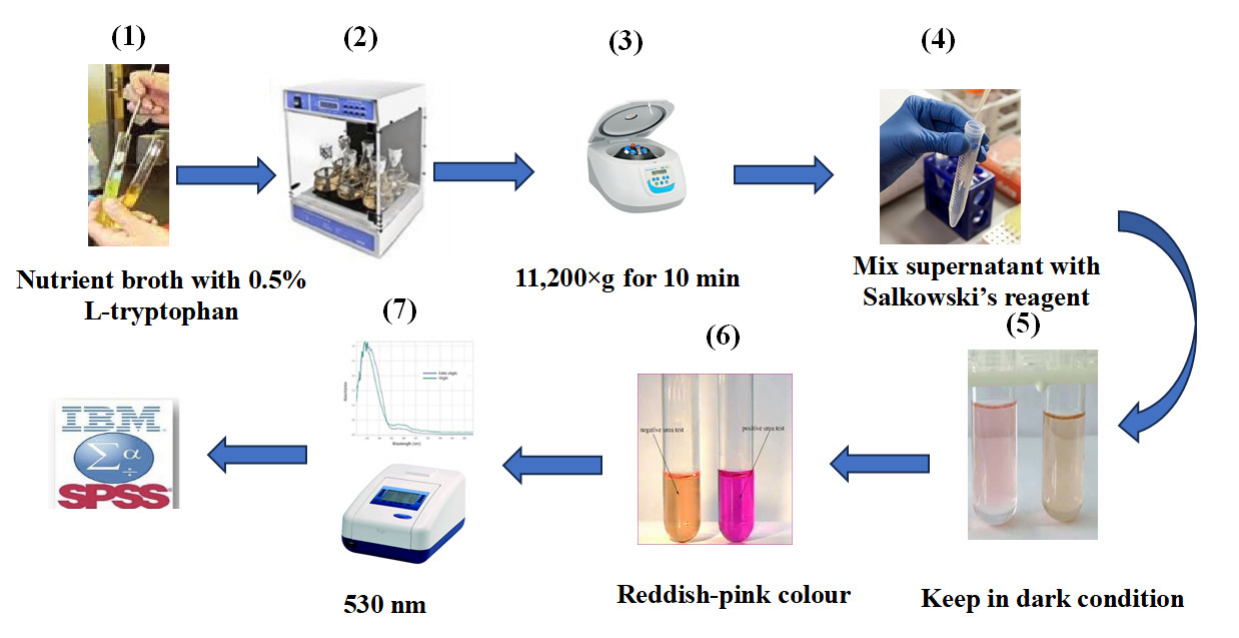
Quantitative estimation of auxin production in halotolerant bacterial isolates for cucumber growth. 1) Inoculate isolates in nutrient broth medium containing 0.5% L-tryptophan. 2) Incubate for 48 h on a rotary shaker. 3) Centrifuge bacterial cultures at 11,200× *g* for 10 min. 4) Mix 1 mL of the supernatant with 1 mL of Salkowski’s reagent. 5) Keep in the dark for 20 min. 6) A reddish-pink color indicates the production of IAA. 7) Measure absorbance at 530 nm.

**E. Measurement of siderophore production**

*Note: Siderophore production was assessed using the CAS shuttle assay method (Castaneda et al., 2005), which is based on the color change of CAS from blue to orange due to iron removal by siderophores.*

1. Culture selected isolates in iron-minimal medium at 30 °C for 48 h (Figure 2.1).

2. Centrifuge the samples at 81,600× *g* for 15 min (Figure 2.2).

3. Add 0.5 mL of CAS reagent to each supernatant from the isolate cultures (Figure 2.3).

4. Incubate the samples at 30 °C for 20 min (Figure 2.4).

5. Iron removal in the presence of siderophores results in a color change from blue to orange (Figure 2.5).

6. Measure color intensity using a spectrophotometer at a wavelength of 630 nm (Figure 2.6).

7. Calculate siderophore production as a percentage using the following formula [22]:

Siderophore production (%) = {(Ar - As) × 100}/Ar

Where: Ar = reference absorbance (CAS solution + uninoculated medium)

As = sample absorbance (CAS solution + cell-free supernatant of the sample)

**Figure 2.**
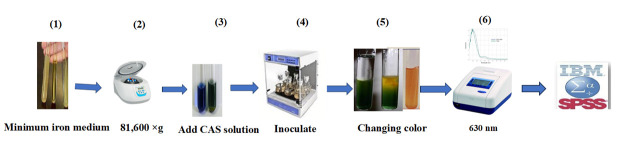
Quantitative estimation of siderophore production in halotolerant bacterial isolates for cucumber growth. 1) Culture selected isolates in minimum iron medium at 30 °C. 2) Centrifuge samples for 15 min at 81,600× *g.* 3) Add CAS solution to the supernatant. 4) Incubate at 30 °C for 20 min. 5) Observe a decrease in the intensity of the blue color. 6) Measure the color intensity at 630 nm.

**F. Measurement of hydrogen cyanide (HCN) production**

**Figure 3.**
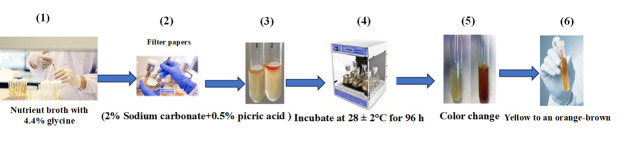
Quantitative estimation of hydrogen cyanide production in halotolerant bacterial isolates for cucumber growth. 1) Grow bacterial isolates in nutrient broth containing 4.4% glycine. 2) Soak filter papers in a solution of 2% sodium carbonate and 0.5% picric acid. 3) Place the soaked filter papers on the surface of the culture medium. 4) Incubate at 28 ± 2 °C for 96 h. 5) Observe for a distinct color change. 6) Note the shift from yellow to orange-brown.

1. Prepare nutrient broth medium containing 4.4% glycine (Figure 3.1).

2. Prepare 3 mL of bacterial suspension (10^6^ CFU/mL) from each isolate.

3. Inoculate bacteria into a 250 mL flask containing 100 mL of the prepared medium at 30 °C for 48 h.

4. Soak a Whatman No. 1 filter paper in a solution of 2% sodium carbonate and 0.5% picric acid and place it over the surface of the culture medium (Figure 3.2–3).

5. Inoculate the samples at 28 ± 2 °C for 96 h (Figure 3.4).

6. Evaluate HCN production by a color change of the filter paper from yellow to orange-brown (Figure 3.5–6).

**G. Investigation of insoluble phosphate solubilization**

1. Prepare solid Picosky medium (Figure 4.1).

2. Inoculate isolates onto solid Picosky medium and incubate at 30 ± 2 °C for five days (Figure 4.2).

3. Assess phosphate solubilization by observing a clear halo around the colony (Figure 4.3).

4. Inoculate phosphate-solubilizing isolates into liquid Picosky medium and shake at 3 rpm for 7 days at 30 ± 2 °C (Figure 4.4–5).

5. Centrifuge at 16,100× *g* for 10 min at 25 °C to remove bacterial cells and solid particles (Figure 4.6).

6. Transfer the clear supernatant from each vial into new vials.

7. Measure absorbance at 820 nm using a spectrophotometer (Figure 4.7).

8. Conduct the quantification of solubilized phosphorus by comparing it with a standard KH_2_PO_4_ curve [23].

9. Select four salt-tolerant isolates with the best growth-promoting characteristics for greenhouse studies.

**Figure 4.**
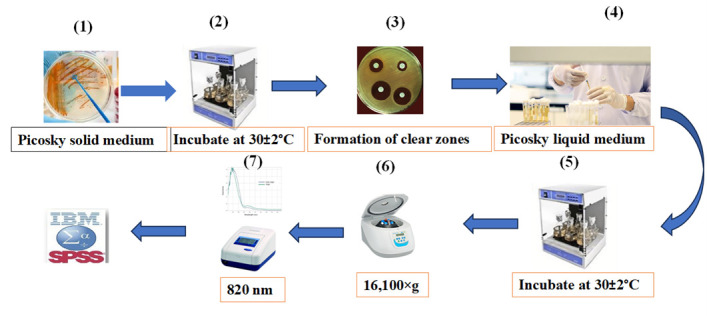
Quantitative estimation of insoluble phosphate solubilization in halotolerant bacterial isolates for cucumber growth. 1) Culture the bacterial isolates on Picosky solid medium. 2) Incubate at 30 ± 2 °C for five days. 3) Monitor the formation of clear zones around the bacterial colonies. 4) Inoculate the isolates into Picosky liquid medium. 5) Incubate for 7 days on a shaker at 150 rpm and 30 ± 2 °C. 6) Centrifuge for 10 min at 16,100× *g* and 25 °C to remove bacterial cells and solid particles. 7) Measure absorbance at 820 nm.

**H. Greenhouse studies**

1. Prepare Royal cultivar cucumber seeds.

2. The seeds undergo surface sterilization for 3 min in a 5% sodium hypochlorite solution, followed by 8–10 rinses with sterile distilled water. Treat them for 3 min with the fungicide captain (Wp50%).

3. Wash the seeds three times with sterile distilled water for 3 min each time.

4. Select four superior isolates (K4, K14, K15, and C8) for evaluation of their growth-promoting potential.

5. Plant eight pre-germinated seeds in each clean pot (21 × 16 ×16 cm) at a depth of 2 cm. Maintain at 28 °C, with 75% relative humidity and a 12/12 h photoperiod (Figure 5.1–2).

6. Inoculate each seed with 3 mL of a bacterial suspension containing 10^8^ cells per mL (Figure 5.3).

7. Arrange the greenhouse experiment as a factorial based on a completely randomized design with two factors: salinity at four levels (control, 4, 8, and 16 dS/m, achieved by adding NaCl to the irrigation water) and five bacterial treatments (no inoculation and separate inoculations with four salt-tolerant bacteria), with three replications. Maintain soil moisture at 70% field capacity.

8. Six weeks after planting, measure vegetative parameters (plant height, fresh and dry weight, and leaf area) (Figure 5.4).

9. Measure seedling height using a graduated ruler with millimeter precision (Figure 5.5).

10. Measure fresh weight using a digital scale accurate to 0.001 g (Figure 5.5).

11. Measure dry weight using a digital scale after drying samples in an oven for 48 h at 72 °C (Figure 5.5).

12. Measure leaf area using a leaf scanner and Photoshop graphic software (Figure 5.5).

**Figure 5.**
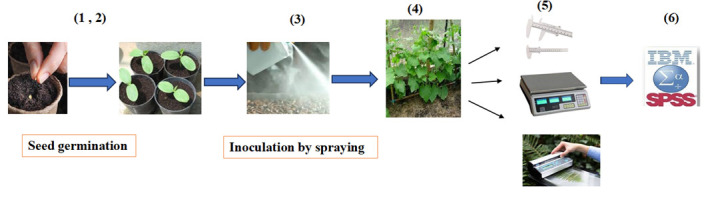
Plant growth-promoting ability of the four superior isolates. 1) Surface-sterilize the cucumber seeds. 2) Plant the seeds in pots at a depth of 2 cm. 3) Add 3 mL of bacterial suspension to each seed. 4) Grow the plants for six weeks. 5) Measure plant height, fresh and dry weight, and leaf area. 6) Conduct data analysis.

## Data analysis

**Figure 6.**
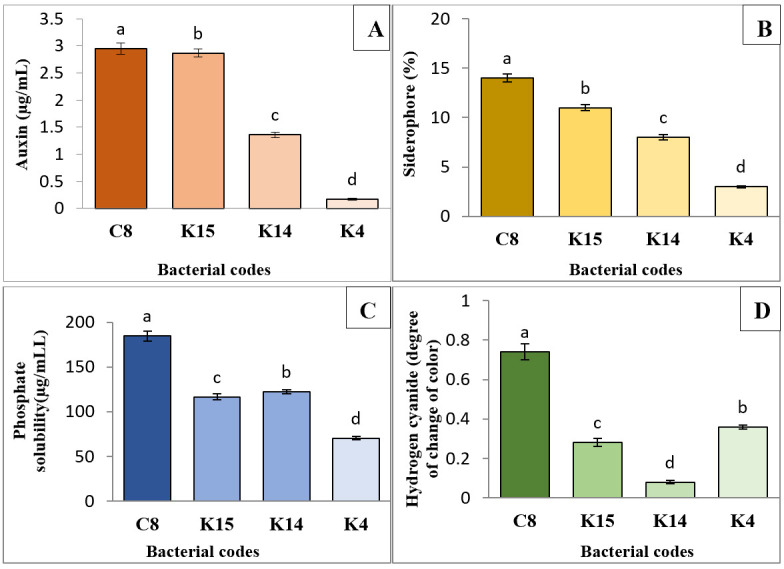
Comparison of mean production of auxin (A), siderophore (B), phosphate solubilization activity (C), and hydrogen cyanide (D) by the four superior isolates isolated from different areas of Jiroft (Iran). (K: Kanar Sandal and C: Karimabad). Significant differences were determined by Duncan’s t-test. *p < 0.05. Treatments sharing the same letter are not significantly different, while those with different letters are statistically distinct. Error bars represent standard error.

Our previous work [24] demonstrated that the superior isolates—K4, K14, K15, and C8—can produce growth-promoting substances (Figure 6). Auxin production by these isolates ranged from 0.17 to 2.95 μg/mL. In addition, all isolates were capable of producing siderophores (Figure 3B), with C8 and K15 yielding the highest amounts at 14% and 11%, respectively (Figure 6B). The phosphate solubilization test results indicated that all four isolates could solubilize water-insoluble inorganic phosphate, with C8 and K14 exhibiting the highest activities at 184.64 and 122.11 μg/mL, respectively (Figure 6C). Regarding hydrogen cyanide production, the C8 isolate produced the highest amount (0.74), while K14 produced the lowest (0.08) (Figure 6D).

**Plant growth-promoting properties of the superior isolates**

Results showed all the superior isolates viz., K4, K14, K15, and C8, were able to produce auxin, hydrogen cyanide, siderophore and they also possessed phosphate solubilization activity. The results of auxin production by isolates are shown in Fig. 4. The amount of auxin production by isolates ranged from 0.17 to 2.95 μg ml^-1^. The highest auxin production capacity was observed for C8 isolate by 2.95 μg ml^-1^, while K15 and K4 isolates had the lowest of auxin production (Figure 6A). Furthermore,The results of siderophore production potentiality of isolates showed that all isolates were positive for siderophore production (Figure 4B). Additionally, C8 and K15 isolates produced the highest amount of siderophore with values of 14% and 11% (Figure 6B). Results of the phosphate solubilization test showed that all four isolates were able to solubilize the water-insoluble inorganic phosphate compound, with C8 and K14 isolates showing the highest phosphate solubilization activities by 184.64 and 122.11 μg ml^−1^, respectively (Figure 6C). In terms of hydrogen cyanide production, C8 isolate was able to produce the highest amount of hydrogen cyanide (0.74) whereas K14 isolate produced the lowest amount (0.08) (Figure 6D).

**Figure 7.**
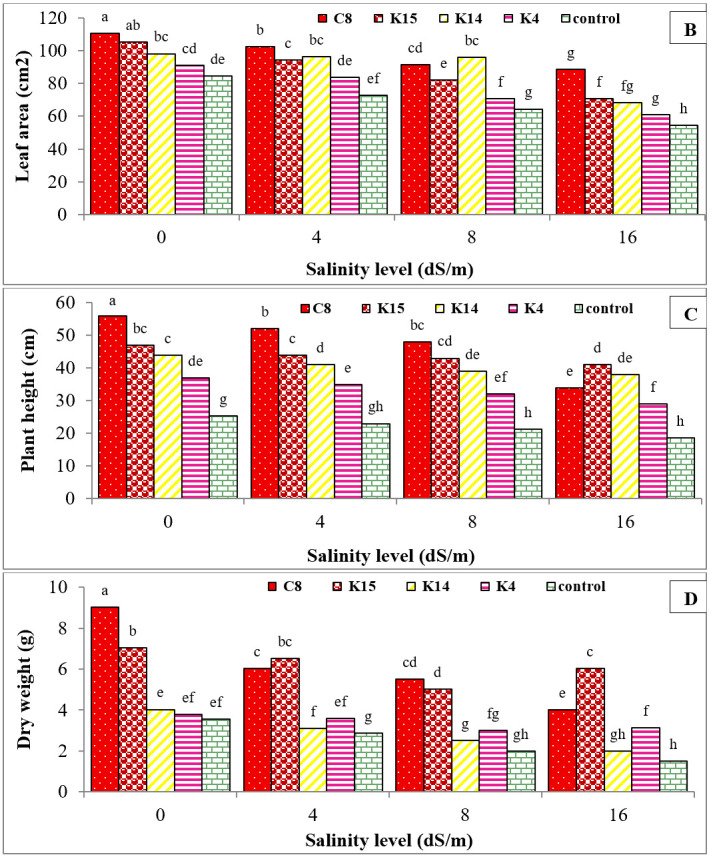
Comparison of mean effect of superior bacterial isolates and their interactions on the fresh weight (A), leaf area (B), plant height (C), and dry weight (D) of cucumber seedlings under salinity stress after six weeks of growth. (K: Kanar Sandal and C: Karimabad). Significant difference was determined by Duncan’s t-test. * p < 0.05.

Exposure to salinity stress at 16 dS/m significantly reduced plant height, fresh and dry weights, and leaf area by 29%, 20%, 42%, and 42%, respectively, compared to the control (Figure 7A, B, C, D). Statistical analysis showed that the selected superior isolates significantly improved all growth parameters of cucumber plants subjected to salinity stress for six weeks, with increases of 41.47% in plant height, 34.97% in fresh weight, 7.49% in dry weight, and 85.07% in leaf area (Figure 7A, B, C, D).

## Validation of protocol

This protocol or a part of it has been used and validated in the following research articles:

- Barazani and Friedman. [20]. Effect of exogenously applied l-tryptophan on allelochemical activity of plant growth promoting rhizobacteria (PGPR). *Journal of Chemical Ecology.*
- Castaneda et al. [25]. A spectrophotometric method to determine the siderophore production by strains of *Pseudomonas fluorescent* in the presence of copper and iron. *Microchemical Journal.*
- Castric. [26]. Hydrogen cyanide, a secondary metabolite of *Pseudomonas aeruginosa*. Canadian Journal of Microbiology.
- Pikovskaya. [27]. Mobilization of phosphorus in soil in connection with vital activity of some microbial species. *Microbiologyal.*

## General notes and troubleshooting

**General notes**

1. Collect soil samples from saline and arid fields to isolate salt and drought-resistant strains.

2. Consider the expiration date when preparing each culture medium.

3. Use fresh isolates for every test step.

4. Include a control group and three replicates for each test.

5. Acquire cucumber seeds from approved sources.

6. Verify that the seeds are healthy and sterile.

7. Maintain consistent storage conditions across all treatments.

8. Utilize the same measuring tools and equipment for all tests.

**Troubleshooting**

**Problem 1:** Seeds have not germinated after three days.

Possible cause: The seeds may be dormant.

Solution: Wait at least seven days, as some seeds can take up to 10 days to germinate, especially in the dark.

**Problem 2:** There is no clear halo around the colony when assessing the phosphate-solubilizing power of isolates.

Possible cause: The culture medium may be old or damaged.

Solution: Use fresh, healthy, and approved culture medium.
